# pIL6-TRAIL-engineered umbilical cord mesenchymal/stromal stem cells are highly cytotoxic for myeloma cells both in vitro and in vivo

**DOI:** 10.1186/s13287-017-0655-6

**Published:** 2017-09-29

**Authors:** Paola Cafforio, Luigi Viggiano, Francesco Mannavola, Eleonora Pellè, Concetta Caporusso, Eugenio Maiorano, Claudia Felici, Francesco Silvestris

**Affiliations:** 1 Department of Biomedical Sciences and Human Oncology, Section of Internal Medicine and Clinical Oncology, University of Bari Aldo Moro, P.za G. Cesare, 11, 70124 Bari, Italy; 2 Department of Biology, University of Bari Aldo Moro, via E. Orabona 4, 70125 Bari, Italy; 3 Department of Emergency and Organ Transplantations, Section of Pathological Anatomy, University of Bari Aldo Moro, P.za G. Cesare, 11, 70124 Bari, Italy

**Keywords:** Multiple myeloma, Umbilical cord mesenchymal stromal/stem cells, TRAIL, Apoptosis

## Abstract

**Background:**

Mesenchymal/stromal stem cells (MSCs) are favorably regarded in anti-cancer cytotherapies for their spontaneous chemotaxis toward inflammatory and tumor environments associated with an intrinsic cytotoxicity against tumor cells. Placenta-derived or TRAIL-engineered adipose MSCs have been shown to exert anti-tumor activity in both in-vitro and in-vivo models of multiple myeloma (MM) while TRAIL-transduced umbilical cord (UC)-MSCs appear efficient inducers of apoptosis in a few solid tumors. However, apoptosis is not selective for cancer cells since specific TRAIL receptors are also expressed by a number of normal cells. To overcome this drawback, we propose to transduce UC-MSCs with a bicistronic vector including the *TRAIL* sequence under the control of IL-6 promoter (*pIL6*) whose transcriptional activation is promoted by the MM milieu.

**Methods:**

UC-MSCs were transduced with a bicistronic retroviral vector (pMIGR1) encoding for green fluorescent protein (GFP) and modified to include the *pIL6* sequence upstream of the full-length human *TRAIL* cDNA. TRAIL expression after stimulation with U-266 cell conditioned medium, or IL-1α/IL-1β, was evaluated by flow cytometry, confocal microscopy, real-time PCR, western blot analysis, and ELISA. Apoptosis in MM cells was assayed by Annexin V staining and by caspase-8 activation. The cytotoxic effect of *pIL6-TRAIL* ^*+*^ *-GFP* ^*+*^-UC-MSCs on MM growth was evaluated in SCID mice by bioluminescence and ex vivo by caspase-3 activation and X-ray imaging. Statistical analyses were performed by Student’s *t* test, ANOVA, and logrank test for survival curves.

**Results:**

*pIL6-TRAIL* ^*+*^ *-GFP* ^*+*^-UC-MSCs significantly expressed TRAIL after stimulation by either conditioned medium or by IL-1α/IL-1β, and induced apoptosis in U-266 cells. Moreover, when systemically injected in SCID mice intratibially xenografted with U-266, those cells underwent within MM tibia lesions and significantly reduced the tumor burden by specific induction of apoptosis in MM cells as revealed by caspase-3 activation.

**Conclusions:**

Our tumor microenvironment-sensitive model of anti-MM cytotherapy is regulated by the axis pIL6/IL-1α/IL-1β and appears suitable for further preclinical investigation not only in myeloma bone disease in which UC-MSCs would even participate to bone healing as described, but also in other osteotropic tumors whose milieu is enriched of cytokines triggering the pIL6.

## Background

Mesenchymal stem cells (MSCs) are presently under intensive investigation aimed not only at elucidating their nature and propensity to generate skeletal-related tissues [1, 2], but also to develop possible cell-based therapies against a number of diseases including cancer [3–5]. In this regard, within a few gene-therapy approaches, MSCs from bone marrow (BM) as well as from adipose tissue (AT) have been modified to enhance their secretory functions in a more targeted fashion either by releasing specific cytokines [6, 7] or as pro-apoptogen molecule producers to reverse the cell growth in both in vitro and in vivo models of solid tumors [8–10]. Consistent work by different groups of investigators showed indeed that both BM-derived and AT-derived MSCs are capable of inducing the tumor shrinkage in xenografted human glioma [11], gastric [12] or pancreatic [13] cancers, as well as in melanoma [14], and that this native anti-tumor cell growth activity may be definitely enhanced with MSCs transduced to express the tumor necrosis factor related apoptosis inducing ligand (TRAIL), namely a pro-apoptogen molecule linking the death receptors (DR) 4 and 5 on cancer cells [15]. In this context, fetal MSCs from umbilical cord (UC) engineered to produce a membrane-TRAIL protein have been reported to efficiently restrain the intracranial glioblastoma growth in mice as an effect of their innate chemotactic tendency to migrate toward the tumor microenvironment while exposing the death ligand to the tumor cells [16].

Based on their fetal tissue derivation, UC-MSCs have thus been focused with high interest to design novel cell-based therapeutic strategies. Besides their native restraining activity on Burkitt’s lymphoma cell proliferation [17], these cells have been demonstrated by ourselves to express a peculiar molecular profile of inhibitory properties on malignant plasma cells in vitro in relation to their secretome which significantly differs from both BM-derived and AT-derived MSCs [18]. On the other hand, placenta-derived MSCs, as a further fetal tissue source, are also capable of exerting spontaneous killing of multiple myeloma (MM) cells both in vitro and in a severe combined immunodeficient (SCID)-rab mice MM model developing osteolytic lesions [19]. In these studies, in addition to the anti-myeloma activity, MSCs were also found capable of repairing the bone loss within the bone lytic lesions in relation to their bone-regenerating constitutive capability [20].

However, since MSCs, particularly BM-MSCs, physiologically support hematopoiesis, it has also been debated whether a MSC-based model of cytotherapy for MM would sustain, rather than suppress, the proliferation of malignant plasma cells [21]. Contrarily to BM-derived and AT-derived MSCs, those from fetal tissues appear resistant to the genomic conditioning by MM cells that drives the molecular potential to support tumor cell proliferation [22], whereas UC-MSCs apparently show a genomic profile of a definite anti-MM killing secretome [18].

Previous work with TRAIL-engineered BM-MSCs or UC-MSCs, however, included transduction of cells with viral vectors allowing the constant expression of the apoptogen molecule by the cell membranes with the potential risk of cell death induction even in normal cells exposing DR4/DR5 receptors. Unselective binding of target cells thus represents a major drawback of this cytotherapy model since the occurrence of liver as well as of other parenchymal damage has already been reported in preliminary studies by Kim et al. [16] when treating glioma in mice with TRAIL-transduced UC-MSCs.

To overcome such a major drawback and generate UC-MSCs transduced to kill MM cells, we thus used a bicistronic vector to regulate *TRAIL* expression only after their molecular cross-talk with soluble factors of the MM tumor microenvironment. During the tumor progression of myeloma within the bone marrow, indeed, both interleukin (IL)-1α and IL-1β secreted by MM cells stimulate the stroma to produce IL-6 [23] through the linkage of the early growth response (EGR)-1 protein to the promoter of *IL-6* (*pIL6*) [24]. Therefore, we transduced the UC-MSCs with a vector containing the full-length cDNA sequence of *TRAIL* under the control of the *pIL6* and we evaluated the potential of *pIL6-TRAIL* ^*+*^ *-GFP* ^*+*^-UC-MSCs to eradicate MM cells both in vitro and in SCID mice bearing intratibial human myeloma.

Results from our study support the effectiveness of an anti-MM cytotherapy approach in terms of selective killing of malignant plasma cells.

## Methods

### Cell cultures

UCs were obtained from parturients at the Obstetrician and Gynecology Department after informed consent approved by the local Ethical Committee of the University of Bari. UC-MSCs were isolated and maintained in alpha-Modified Eagle’s Medium (MEM) (Gibco, Life Technol., Lofer, Austria) and, at the second passage, were used for retroviral transduction. The U-266 MM cell line (DSMZ, Braunschweig, Germany) was grown in complete Roswell Park Memorial Institute (RPMI)-1640 medium (Gibco), whereas HEK293T, a human embryonic kidney cell line (Sigma Aldrich, St. Louis, MO, USA), was cultured in Dulbecco’s modified Eagle’s medium (DMEM) (Gibco).

### *TRAIL* transduction of UC-MSCs

**Fig. 1:**
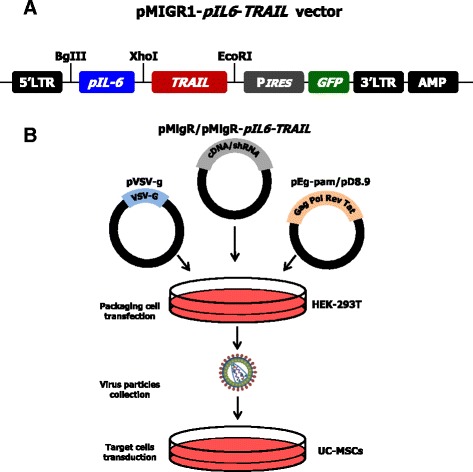
Structure of pMIGR1 vector and steps for UC-MSC transduction. **a** Structural construction of the bicistronic retroviral vector including both *TRAIL* and *GFP* sequences controlled by the *IL-6* promoter. P*IRES* sequence was inserted to codify two different proteins from a single mRNA. **b** Sequential phases of multiple cell transfection, viral particle enrichment, and final transduction of UC-MSCs. GFP green fluorescent protein, MSC mesenchymal/stromal stem cell, pIL6 interleukin-6 promoter, P*IRES* poliovirus internal ribosome entry site, TRAIL tumor necrosis factor related apoptosis inducing ligand, UC umbilical cord

To generate *TRAIL*-transduced UC-MSCs, we adopted a bicistronic retroviral vector (pMIGR1) from a murine stem cell virus encoding for the green fluorescent protein (*GFP*) gene. This vector was modified to include the *pIL6* sequence upstream of the full-length human *TRAIL* cDNA (Fig. 1a). Briefly, a 315-nucleotide fragment of human *pIL6* (nucleotides –303 to +12, Ensembl ENSG00000136244), obtained from genomic DNA by cutting with restriction enzymes for *Bgl*II and *Xho*I sites, was amplified in polymerase chain reaction (PCR) by dedicated primers (forward, 5′-GAATTAGATCTTCAAGACATGCCAAAGTGC-3′; and reverse, 5′-GCCATCCTCGAGGGCAGAATGAGCCTCA-3′). Full-length human *TRAIL* gene (NM_003810.2) was amplified from cDNA using Expand High Fidelity Taq (Roche, Indianapolis, IN, USA) by primers containing *Xho*I (forward 5′-GCCCTCGAGGATGGCTATGATGGAGGTCCA-3′) and *Eco*RI (reverse 5′-GCGGAATTCCTTAGCCAACTAAAAAGGCCCC-3′) sites. Both PCR products were digested by *Xho*I and ligated with each other to generate a single insert. Thus, the *pIL6*-*TRAIL* was cloned into pMIGR1 at *Bgl*II and *Eco*RI sites, and defined as *pIL6-TRAIL* ^*+*^ *-GFP* ^*+*^-pMIGR1 vector, whereas the empty *GFP* ^*+*^-pMIGR1 vector was used as control.

Retroviruses were produced by cotransfection of HEK293T cells with both pMIGR1 construct and the packaging plasmids, namely pΔ8.9 and pVSV-G, using XTreme Gene 9 DNA transfection Reagent (Roche). HEK293T retrovirus-enriched supernatants were collected 48 h after transfection and concentrated by ultracentrifuge at 17,000 rpm (SW28 rotor, Optima LE80K Ultracentrifuge; Beckman, Brea, CA, USA) for 2 h at 4 °C (Fig. 1b). Thus, UC-MSCs were transduced by virus-containing media from either *pIL6-TRAIL* ^*+*^ *-GFP* ^*+*^-HEK293T or *GFP* ^*+*^-HEK293T (both ∼ 1 × 10^6^ transducing units/ml) and 4 μg/ml polybrene (Sigma-Aldrich). The transfected UC-MSCs were distinguished as *pIL6-TRAIL* ^*+*^ *-GFP* ^*+*^-UC-MSCs and *GFP* ^*+*^-UC-MSCs respectively, and expanded until the seventh passage.

### TRAIL expression and modulation in transfected UC-MSCs

After transfection, UC-MSCs were investigated by flow cytometry (FACScanto; Becton-Dickinson, San Diego, CA, USA) for GFP fluorescence to define the efficiency of the vector insertion. The transfected UC-MSCs were then sorted for GFP expression by FACS Aria III (Becton Dickinson, Milan, Italy) to obtain homogeneous populations to be used in subsequent experiments.

Basal levels of TRAIL and further expression on *pIL6-TRAIL* ^*+*^ *-GFP* ^*+*^-UC-MSCs by U-266 supernatant, and by 10 ng/ml of IL-1α and IL-1β, were investigated by flow cytometry, confocal microscopy, quantitative (q)PCR, and western blot (WB) analysis.

TRAIL flow cytometry analysis was assessed using specific phycoerythrin (PE)-conjugated monoclonal (Mo) antibody (Ab) (Abcam, Cambridge, UK) in triplicate with isotype control. Data were reported as both the percentage of TRAIL-positive cells and the mean fluorescence intensity (MFI) ratio as described previously [25].

Furthermore, TRAIL expression by *pIL6-TRAIL* ^*+*^ *-GFP* ^*+*^-UC-MSCs was also analyzed by confocal microscope and NIS element software (C2plus; Nikon Instr., Lewisville, TX, USA). Transfected UC-MSCs were incubated with unconjugated anti-human TRAIL rabbit polyclonal Ab (Cell Signaling, Danvers, MA, USA) and then with anti-rabbit fluorescein isothiocyanate (FITC) (Sigma). The samples were counterstained by tetramethylrhodamine (TRITC)-conjugated phalloidin (Life Technologies) to visualize F-actin and by Hoechst 33342 (Sigma Aldrich) for nuclear staining.

In addition, to assess the TRAIL expression by qPCR, total RNA was extracted by RNeasy kit (Qiagen, Hilden, Germany), and 500 ng was reverse-transcribed by IScript cDNA synthesis kit (Bio-Rad, Hercules, CA, USA). cDNA was then amplified by Fast SYBR Green Master Mix and the StepOne Plus Real Time PCR (Life Technologies Inc., Carlsbad, CA, USA) using the specific primers for *TRAIL*: forward, 5′-GCTCTGGGCCGCAAAAT-3′; and reverse, 5′-TGCAAGTTGCTCAGGAATGAA-3′. Data were normalized on glyceraldehyde-3-phosphate dehydrogenase (*GAPDH*) levels and *TRAIL* amounts were detected as fold change with respect to basal condition.

Also, the protein was evaluated by WB analysis using polyclonal anti-human TRAIL Ab (Abcam) and ECL reagent (Bio-Rad), and then visualized by the UVIchemi (UVItec, Cambridge, UK) imaging system using UVI-1D quantification software. Expression levels were calculated as mean ± 3 standard deviations (SDs) of the optical density (OD) ratio between TRAIL and housekeeping GAPDH in three different experiments. Finally, soluble TRAIL was also measured in supernatants of *pIL6-TRAIL* ^*+*^ *-GFP* ^*+*^-UC-MSCs after treatment with U-266 conditioned medium, or IL-1α and IL-1β, by dedicated ELISA kit (Abcam).

### In vitro apoptosis of U-266 cells

To investigate the pro-apoptogen potential of *pIL6-TRAIL* ^*+*^ *-GFP* ^*+*^-UC-MSCs toward U-266 cells, we carried out cocultures at 1:2 ratio and evaluated the cytotoxicity at 24 h by Annexin V-FITC/propidium iodide (PI) staining (eBioscience, Bender MedSystems GmbH, Vienna, Austria) using the FACScanto. The U-266 population was gated based on forward scatter (FSC) and side scatter (SSC) parameters.

Specificity of apoptosis of U-266 cells following the TRAIL cross-talk was analyzed by caspase-8 activation using the CaspGLOW™ Fluorescein Active Caspase-8 Staining Kit (eBioscience, Bender MedSystems GmbH) and the active caspase-8 was evaluated by flow cytometry.

### In vivo functional studies

To investigate the activity of transfected UC-MSCs against MM cells in vivo, we generated stably transduced bioluminescent U-266 cells (Red-Luc^+^U-266) using RediFect™ lentiviral particles containing red-shifted firefly luciferase (*Luciola italica*) transgene (Perkin Elmer, Waltham, MA, USA). Briefly, U-266 cells were seeded into a 24-well plate and were incubated with 10^6^ viral particles for 24 h. Transduced cells were selected and expanded in medium containing 1 μg/ml puromycin for 4 weeks. Luciferase expression by transduced U-266 was assayed using IVIS Lumina SIII (Perkin Elmer) by adding d-luciferin potassium salt (Perkin Elmer) to the cultures.

Anti-MM activity of *pIL6-TRAIL* ^*+*^ *-GFP* ^*+*^-UC-MSCs was investigated in 6–8-week-old NOD.CB17-Prkdcscid/J mice (Charles River, Milan, Italy) in line with the rules and institutional guidelines of the Italian Ministry of the Health. For this, 42 mice were intratibially (IT) injected with 2 × 10^5^ Red-Luc^+^U-266 cells and the tumor engraftment was evaluated by luminescence imaging 3 days after inoculation. Briefly, anesthetized mice were intraperitoneally administered with luciferin (150 mg/kg) and the tumor luminescence was captured 10 min after injection. Regions of interest encompassing the area of signal were defined using Living Image software and the total photons per second (photons/s) were recorded. The MM-bearing mice were then randomly divided into three groups and injected intracardially (IC) as follows: phosphate buffered saline (PBS) for the control group (*N* = 14); 2.5 × 10^5^ *GFP* ^*+*^-UC-MSCs (*N* = 14); and 2.5 × 10^5^ *pIL6-TRAIL* ^*+*^ *-GFP* ^*+*^-UC-MSCs (*N* = 14). Three mice from each group were sacrificed after 12 and 48 h to evaluate the distribution of UC-MSCs and MM cell apoptosis within tibiae. In addition, the tumor burden was evaluated by luminescence imaging in six mice of each condition at different times up to 30 days after the treatment with *pIL6-TRAIL* ^*+*^ *-GFP* ^*+*^-UC-MSCs and *GFP* ^*+*^-UC-MSCs as control. The tumor growth rate in each group of MM-bearing mice was expressed as the relative fold increase of tumor volume calculated as the ratio of total photon flux at various time points with respect to basal condition. All animals were ultimately sacrificed at 30 days for ethical reasons.

### Ex vivo measurement of *pIL6-TRAIL*^*+*^*-GFP*^*+*^-UC-MSC activity against MM

Several tissue samples, including heart, lung, spleen, liver, and kidney from MM-bearing mice sacrificed at 12 and 48 h, were fixed in formaldehyde and embedded in paraffin, whereas explanted tibiae were decalcified in formic acid and included in paraffin.

PCR for the *GFP* insert was performed to reveal the *pIL6-TRAIL* ^*+*^ *-GFP* ^*+*^-UC-MSC distribution in mice. DNA was extracted from all tissues using the AllPrep DNA/RNA FFPE Kit (Qiagen). Briefly, 100 ng template DNA was used to perform PCR using the HotstarTaq Master Mix (Qiagen) and 0.3 μM of primers, for 35 cycles with annealing temperatures of 60 °C (GFP) or 55 °C (β-actin). Primer sequences for *GFP* were forward 5′GTGCTTCAGCCGCTACCC-3′ and reverse 5′-TGTCGGCCATGATATAGACGTTG-3′, whereas for *β-actin* they were forward 5′-ACGGGGTCACCCACACTGTGC-3′ and reverse 5′-CCGCTCGTTGCCAATAGTGATGA-3′.

To evaluate the intratibiae MM cell apoptosis, sections 3 μm thick were stained with hematoxylin–eosin and in parallel for active caspase-3 by a specific anti-human mouse MoAb (MyBiosource, San Diego, CA, USA). The test was completed by EnvisionFlex kit (DakoCytomation, Santa Clara, CA, USA) according to the manufacturer’s instructions. All samples were then examined under light microscopy (Olympus Bx61; Shinjuku, Tokyo, Japan). To visualize the macroscopic effect of our model, we completed radiography evaluations of tibiae. Briefly, animals were euthanized by carbon dioxide and X-ray scans were taken at 20 kV and 25 mAs for 5 s using a mammographic device (Model Flat E; Metaltronica, Rome). Films from the three groups were inspected comparatively for visible bone lesions that were carefully measured for their bone devastation size (mm^2^) (ImageJ software, version 1.45; NIH, Bethesda, MD, USA).

### Statistical analysis

Results were shown as mean ± SD of experimental triplicates. Statistical analyses were completed by Microsoft® Excel (Microsoft, Inc., Redmond, WA, USA) and GraphPad Software (GraphPad Software, San Diego, CA, USA). Significance between differences in Kaplan–Meier survival curves were generated using MedCalc 12.7.0.0 software. For the Kaplan–Meier analyses, survival curves were compared using the logrank test. Student’s *t* test was used to compare two groups while comparisons between multiple groups (*n* > 2) were performed by ANOVA and differences were considered significant with *p* < 0.05.

## Results

### *pIL6-TRAIL*^*+*^*-GFP*^*+*^-pMIGR1 vector construction and UC-MSC transduction

pMIGR1 bicistronic retroviral vector containing poliovirus internal ribosome entry site (P*IRES*) and *GFP* sequences was modified to express full-length TRAIL under the control of *pIL6* (Fig. 1a). *pIL6-TRAIL* construct was obtained by ligation of the relative PCR products in the *Xho*I restriction site and subsequently cloned between the *Bgl*II and *Eco*RI sites on pMIGR1. Thus, modified pMIGR1 construct and viral packaging plasmids were transfected in HEK293T competent cells and the culture supernatant containing viral particles bearing *pIL6-TRAIL* ^*+*^ *-GFP* ^*+*^-pMIGR1 vector was used to infect UC-MSCs (Fig. 1b). pMIGR1 wildtype construct was used as control empty vector.

### TRAIL expression in transduced UC-MSCs

**Fig. 2:**
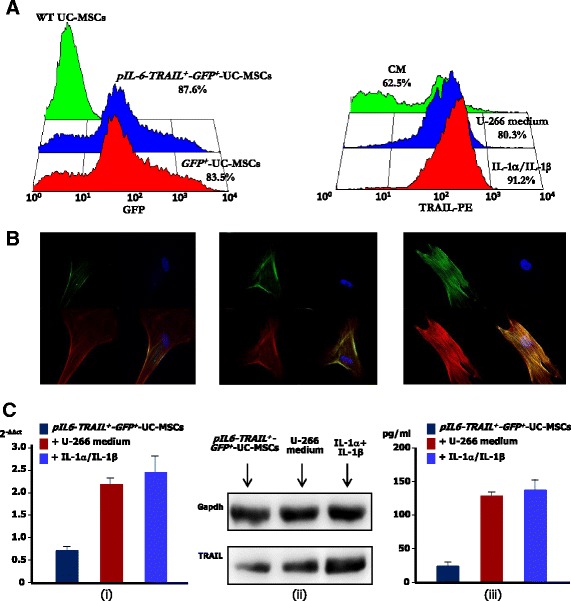
TRAIL expression and modulation in *pIL6-TRAIL* ^*+*^ *-GFP* ^*+*^-UC-MSCs. **a** Left: transfection efficiency of UC-MSCs evaluated by flow cytometry for GFP fluorescence. Wildtype UC-MSCs were negative as compared to *GFP* ^*+*^-UC-MSCs (red histogram: 83.5%) in a similar amount as for *pIL6-TRAIL* ^*+*^ *-GFP* ^*+*^-UC-MSCs (blue histogram: 87.6%). Right: FACS analysis revealed constitutive TRAIL expression in transduced UC-MSCs (green histogram: 62.5%) that was significantly enhanced by the conditioned medium from U-266 cells (blue histogram: 80.3%) as well as in response to IL-1α/IL-1β (red histogram: 91.2%). **b** Confocal microscopy of *pIL6-TRAIL* ^*+*^ *-GFP* ^*+*^-UC-MSCs confirmed the upregulation of TRAIL (FITC, green) induced by either U-266 supernatant or IL-1α/IL-1β treatment. Phalloidin staining was used to show actin (TRITC-red); nuclei were counterstained with Hoechst 33342 (blue). Magnification: 100×. **c** Evaluation of TRAIL expression in transduced cells by RT-qPCR, western blot analysis, and ELISA. (i) After either U-266 or IL-1α/IL-1β treatment, TRAIL mRNA levels in *pIL6-TRAIL* ^*+*^ *-GFP* ^*+*^-UC-MSCs increased up to 2.2-fold and 2.5-fold compared to basal condition. Data are relative amount of mRNA expression normalized to GAPDH and are presented as mean ± SD of three independent experiments. *p < 0.05. (ii) Western blot analysis of lysates from transduced cells showing increased levels of TRAIL protein after U-266 medium or IL-1α/IL-1β conditioning. GAPDH was used as loading control. (iii) ELISA measurement of soluble TRAIL in supernatants of *pIL6*-*TRAIL* ^+^-*GFP* ^+^-UC-MSCs after 24 h of treatment with both U-266 conditioned medium and IL-1α/IL-1β. In both instances the increase was significant as compared to control cells (*p* < 0.05). GFP green fluorescent protein, MSC mesenchymal/stromal stem cell, pIL6 interleukin-6 promoter, TRAIL tumor necrosis factor related apoptosis inducing ligand, UC umbilical cord, WT wildtype

The efficiency of transfection was evaluated by GFP expression in flow cytometry. As shown in Fig. 2a (left), GFP was largely detected in 83.5% of *GFP* ^*+*^-UC-MSCs and 87.6% of *pIL6-TRAIL* ^*+*^ *-GFP* ^*+*^-UC-MSCs, whereas wildtype UC-MSCs were negative as expected. After cell sorting we obtained *GFP* ^*+*^-UC-MSCs and *pIL6-TRAIL* ^*+*^ *-GFP* ^*+*^-UC-MSCs with purity of 99.0% and 99.5% respectively. These last cell populations were expanded and used for subsequent experiments.

The expression of TRAIL, both constitutively and following the variation by U-266 conditioned medium, or by IL-1α and IL-1β, in *pIL6-TRAIL* ^*+*^ *-GFP* ^*+*^ *-*UC-MSCs is depicted by flow cytometry histograms in Fig. 2a (right). As shown, TRAIL was expressed in 62.5% transduced cells in basal conditions suggesting that *pIL6-TRAIL* ^*+*^ *-GFP* ^*+*^ *-*UC-MSCs were able to maintain the constitutive protein production including cytokines that activate *pIL6* [18]. However, when treated with the U-266 conditioned medium, *pIL6-TRAIL* ^*+*^ *-GFP* ^*+*^-UC-MSCs significantly enhanced their TRAIL expression up to 80.3% of positive cells (blue histogram), which was confirmed up to 91.2% of positive population by IL-1α and IL-1β (red histogram). Moreover, TRAIL MFI was 74.6 on *pIL6-TRAIL* ^*+*^ *-GFP* ^*+*^-UC-MSCs after stimulation with the U-266 conditioned medium that was significantly increased compared to basal condition MFI (44.7). The TRAIL upregulation was also confirmed after stimulation by IL-1α and IL-1β (MFI = 70.7). The increased TRAIL expression was shown on representative pictures by confocal microscopy. Figure 2b depicts the enriched TRAIL fluorescent signal on *pIL6-TRAIL* ^*+*^ *-GFP* ^*+*^-UC-MSCs stimulated with U-266 conditioned medium (middle), or in response to IL-1α/IL-1β (right) with respect to basal condition (left).

Furthermore, as shown in Fig. 2c, qPCR of TRAIL mRNA confirmed the stimulation of *pIL6-TRAIL* ^*+*^ *-GFP* ^*+*^-UC-MSCs by either U-266 conditioned medium or IL-1α and IL-1β, since the TRAIL gene expression significantly increased up to 2.2-fold and 2.5-fold respectively as compared to basal condition (Fig. 2ci). This was also confirmed by WB analysis that revealed the increase of TRAIL protein after stimulation. The OD ratio of *pIL6-TRAIL* ^*+*^ *-GFP* ^*+*^-UC-MSCs treated with both U-266 conditioned medium and IL-1α/IL-1β, indeed, was 2.67 ± 1.4 and 3.72 ± 2.1 respectively, compared to untreated cells (*p* < 0.005 in all instances) (Fig. 2cii).

Finally, we also demonstrated the production of soluble TRAIL by transduced UC-MSCs. In fact, ELISA detected minimal mean levels of the ligand (25 pg/ml) in basal condition which, however, was significantly increased in 24-h treated cells up to 140 pg/ml after stimulation with both conditioned medium and IL-1α/IL-1β (*p* < 0.005 in all instances) (Fig. 2ciii).

These results suggested that following the transduction of UC-MSCs with the *pIL6-TRAIL* ^*+*^ *-GFP* ^*+*^ *-*pMigr vector, these cells significantly enhanced their TRAIL expression as both membrane-bound and soluble protein after their activation by *pIL6*.

### *pIL6-TRAIL*^*+*^*-GFP*^*+*^-UC-MSCs are apoptosis effectors in U-266 cells

**Fig. 3:**
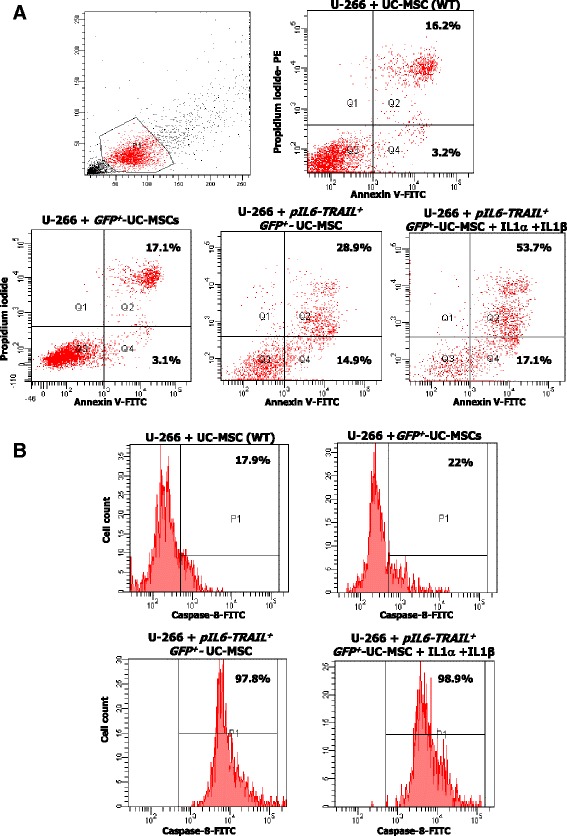
In vitro apoptosis of U-266 cells induced by *pIL6-TRAIL* ^*+*^ *-GFP* ^*+*^-UC-MSCs. **a** Apoptosis in U-266 cells by transduced UC-MSCs was measured by Annexin V/PI staining using flow cytometry. Representative dot plots revealed that the apoptosis extent was significantly increased (43.8%) after 24 h of coculture with *pIL6-TRAIL* ^*+*^ *-GFP* ^*+*^-UC-MSCs with respect to control UC-MSCs (19.4%) and *GFP* ^*+*^-UC-MSCs (20.2%). The effect was also enhanced when IL-1α and IL-1β were added to the cocultures (70.9% of U-266 cell apoptosis). **b** Active caspase-8 in U-266 cells as signature of TRAIL-induced apoptosis was measured by flow cytometry after 24 h of coculture with UC-MSCs, *GFP* ^*+*^-UC-MSCs, and *pIL6-TRAIL* ^*+*^ *-GFP* ^*+*^-UC-MSCs. This representative experiment depicts the activity of caspase-8 in 97.8% of U266 cells cocultured with *pIL6-TRAIL* ^*+*^ *-GFP* ^*+*^-UC-MSCs as compared to 17.9% of control UC-MSCs, and 22% of *GFP* ^*+*^-UC-MSCs. Such high levels of active caspase-8 were not further modified by supplementing the cultures with IL-1α/IL-1β (98.9% of positive cells). GFP green fluorescent protein, MSC mesenchymal/stromal stem cell, pIL6 interleukin-6 promoter, TRAIL tumor necrosis factor related apoptosis inducing ligand, UC umbilical cord, WT wildtype

The capability of *pIL6-TRAIL* ^*+*^ *-GFP* ^*+*^-UC-MSCs to kill MM cells was evaluated in vitro by Annexin V/PI staining. In 24-h coculture, the apoptosis extent of U-266 cells was 50.4% ± 6.6 as compared to control wildtype UC-MSCs (19.4% ± 3.4) or to *GFP* ^*+*^-UC-MSCs (20.2% ± 2.3). When IL-1α and IL-1β were added to the cocultures, we observed an increase of the programmed cell death associated to the additional expression of TRAIL by *pIL6* (70.8% ± 6.5) (*p* < 0.01). Figure 3a shows a representative pattern of U-266 apoptosis induced by TRAIL-transduced UC-MSCs.

To assess the molecular pathway of apoptosis in U-266 cells we measured their levels of active caspase-8. Figure 3b shows these results. As depicted, the flow cytometric analysis of U-266 cells cocultured by *pIL6-TRAIL* ^*+*^ *-GFP* ^*+*^-UC-MSCs suggested a significant enrichment of active caspase-8 up to 97.8% ± 0.7 of this population (*p* < 0.05). The addition of IL-1α and IL-1β was ineffective in changing the active caspase-8 levels (98.9% ± 0.6) as compared to the same cells cultured with UC-MSCs or *GFP* ^*+*^-UC-MSCs (17.9% ± 3.3 and 22.0% ± 2.7 respectively) (*p* < 0.01).

### *pIL6-TRAIL*^*+*^*-GFP*^*+*^-UC-MSCs exert anti-MM activity in vivo

**Fig. 4:**
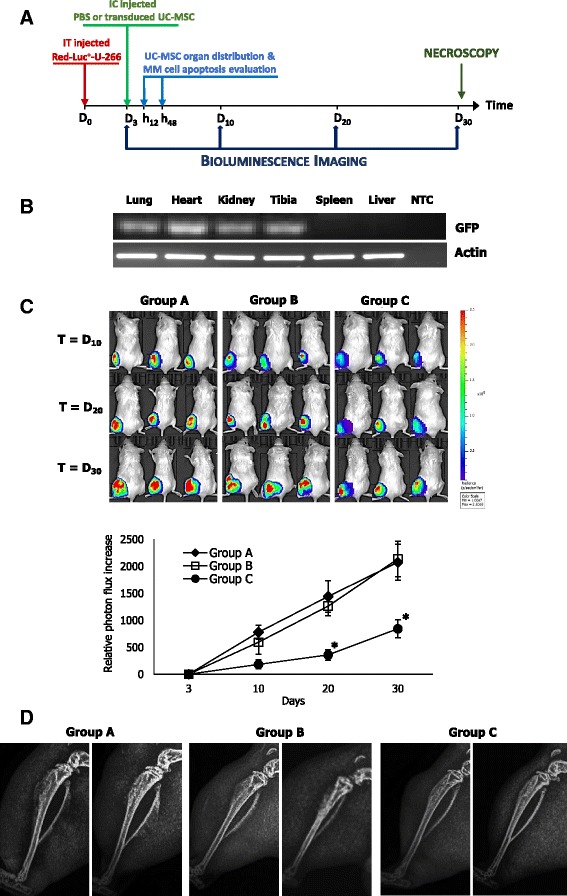
In vivo effect of *pIL6-TRAIL* ^*+*^ *-GFP* ^*+*^ *-*UC-MSCs on MM growth. **a** Timeline elucidating the experimental design of SCID mice xenografted intratibially (IT) with 2 × 10^5^ Red-Luc^+^U266 cells (D_0_), followed after 3 days (D_3_) by injection intracardially (IC) with PBS (group A), 2.5 × 10^5^ *GFP* ^*+*^-UC-MSCs (group B), or 2.5 × 10^5^ *pIL6-TRAIL* ^*+*^ *-GFP* ^*+*^-UC-MSCs (group C) (*N* = 14 for each group). Three mice for each group were sacrificed randomly, respectively at 12 h (h_12_) and 48 h (h_48_) after injection IC for ex vivo evaluation of UC-MSC distribution and tumor apoptosis. The remaining mice were investigated at defined time points (D_3_, D_10_, D_20_ and D_30_) by bioluminescence imaging with IVIS Lumina. **b** The biodistribution of *pIL6-TRAIL* ^*+*^ *-GFP* ^*+*^-UC-MSC, revealed by PCR analysis for *GFP* after injection IC, confirmed the presence of these cells in tibiae as well as in lung, heart, and kidney, while transduced cells were not observed in spleen and liver. Actin was used as loading control. **c** Representative bioluminescence images at different time points of MM-bearing mice, injected IC with PBS (group A), *GFP* ^*+*^-UC-MSCs (group B), or *pIL6-TRAIL* ^*+*^ *-GFP* ^*+*^-UC-MSCs (group C). The color scale ranged from blue (just higher than background noise; set to 1 × 10^7^ photons/s/cm^2^/sr) to red (at least 2.5 × 10^8^ photons/s/cm^2^/sr). Quantitative analysis of tumor growth in mice was assessed by Living Image Software. Data represent the relative increase of median photon flux (photon/s) within ROI areas in each group at different time points. Tumor growth was timely reduced in mice treated with *pIL6-TRAIL* ^*+*^ *-GFP* ^*+*^ *-*UC-MSCs as compared to both PBS and *GFP* ^*+*^-UC-MSC treated groups (*p* < 0.03). Error bars represent the standard error of the mean (SEM); *p value calculated by Student’s *t* test. **d** Hindlimb radiographs of MM bearing mice was performed 27 days after injection IC of transduced UC-MSCs or PBS. Representative images of the three experimental groups show a minor expansion of the osteolytic lesions in mice systemically injected with *pIL6*-*TRAIL* ^+^-*GFP* ^*+*^- UC-MSCs compared to those of groups A and B. GFP green fluorescent protein, MM multiple myeloma, MSC mesenchymal/stromal stem cell, PBS phosphate buffered saline, UC umbilical cord, NTC no template control

We generated stably transduced bioluminescent Red-Luc^+^U-266 to monitor the tumor growth in mice. The timeline of in vivo experiments is reported in Fig. 4a. Forty-two mice were implanted IT with Red-Luc^+^U-266 cells and, after 3 days, the tumor engraftment was evaluated by bioluminescence imaging. Recorded total photon flux at day 3 in all animals was variable, ranging from 3.5 × 10^6^ to 5 × 10^7^ (photon/s) with a mean of 2.64 × 10^7^ ± 2.16 × 10^7^. Thus, mice distributed in the groups were injected IC with PBS (group A), *GFP* ^+^-UC-MSCs (group B), or *pIL6-TRAIL* ^*+*^ *-GFP* ^*+*^-UC-MSCs (Group C). Their drop-out [26], representing the rate of dead animals following the ventricle injection failure, was approximately 14%.

To investigate the organ distribution of transduced UC-MSCs in mice, we analyzed tissues isolated at 12 h after injection IC. PCR analysis for *GFP* confirmed the presence of *pIL6-TRAIL* ^*+*^ *-GFP* ^*+*^-UC-MSCs in tibiae as well as in lung, heart, and renal glomeruli as described previously [27], while transduced cells were not detected in spleen and liver (Fig. 4b). However, there was no evidence of toxicity on healthy tissues evaluated at the experimental endpoint (data not shown).

The MM burden was evaluated by bioluminescence in mice of each group at experimental time points (10, 20, 30 days), as depicted by representative images in Fig. 4c (upper). The quantitative analysis (Fig. 4c, lower), calculated as the ratio of total photon flux at various time points with respect to basal condition, showed a decrease of tumor growth, although not significant, already on D_10_ in group C compared to groups A and B (relative fold increase: group C = 185 ± 80; group A = 778 ± 131; group B = 595 ± 221). At D_20_ the differences between groups were increased, becoming significant (relative fold increase: group C = 360 ± 96; group A = 1442 ± 288; group B = 1264 ± 182), and this effect was maintained at the endpoint (relative fold increase: group C = 844 ± 167; group A = 2076 ± 332; group B = 2136 ± 325) (*p* < 0.03 in each instance).

Bone devastations induced in vivo by MM cells were also evaluated by X-ray imaging. The average size of the osteolytic areas measured on X-ray films of tibiae in group A and B mice was apparently larger and displaying blown cortical bone (mean value 2.5 ± 0.35 mm^2^), as compared to that observed in *pIL6-TRAIL* ^*+*^ *-GFP* ^*+*^-UC-MSC-treated mice (0.96 ± 0.39 mm^2^) (*p* < 0.02) (Fig. 4c).

**Fig. 5:**
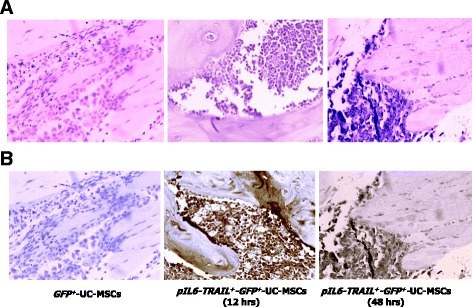
Ex vivo immunohistochemical detection of MM cell apoptosis. **a** Hematoxylin–eosin staining of tibia sections in mice xenografted IT shows the plasma cell bone expansion. As can be seen, U-266 cells are accumulated within resorptive lacunae where they appear strictly to the resorbed bone. **b** Immunostaining of active caspase-3 showed a high extent of apoptosis in tibiae sections of mice injected systemically with *pIL6-TRAIL* ^*+*^ *-GFP* ^*+*^-UC-MSCs after 12 and 48 h. Active caspase-3 was undetectable in control mice treated with *GFP* ^*+*^-UC-MSCs. GFP green fluorescent protein, MSC mesenchymal/stromal stem cell, pIL6 interleukin-6 promoter, TRAIL tumor necrosis factor related apoptosis inducing ligand, UC umbilical cord

Finally, to demonstrate MM cell apoptosis in tibia samples, we measured the active caspase-3 by immunohistochemistry. Figure 5a shows the enrichment of plasma cells evidenced by hematoxylin–eosin staining in the bone matrix of mice tibiae in each group. As shown, plasma cells are accumulated within resorptive lacunae where they appear strictly adjacent to the resorbed bone. Also, the active isoform of caspase-3 was detected already after 12 h from injection IC of *pIL6-TRAIL* ^*+*^ *-GFP* ^*+*^-UC-MSCs and this effect persisted up to 48 h (Fig. 5b). On the contrary, the sections of mice inoculated with *GFP* ^*+*^-UC-MSCs were negative for active caspase-3.

These data suggest that *pIL6-TRAIL* ^*+*^ *-GFP* ^*+*^-UC-MSCs were able to remarkably inhibit the tumor burden and ultimately restrain the bone devastation by U-266 cells.

## Discussion

Novel anti-cancer cytotherapies are presently under intensive investigation and MSCs have potential as cell vehicles for targeted delivery and/or local production of cytotoxic molecules for tumor cells. Besides their large bioavailability and easy recruitment from the human body, the suitability of these cells in fighting cancers is based on their tropism toward inflamed sites including the tumor microenvironment, as well as on their proclivity to be genetically engineered with gene sequences encoding for anti-tumor biological agents [28, 29]. In this context, we have demonstrated previously that UC-MSCs constitutively migrate in vitro toward myeloma cells and express a defined anti-MM secretome [18]. Here, we provide further evidence that, once engineered to express TRAIL in the presence of malignant plasma cells, UC-MSCs specifically induce apoptosis in these cells both in vitro and in vivo in SCID mice bearing human MM. The selective activity against MM cells is related to the control of TRAIL production by *pIL6* which is inserted before the *TRAIL* sequence within the vector, thus rendering suitable this cell-based approach of anti-MM treatment for future translation in human studies.

Previous models of cytotherapies with MSCs in MM included placenta-derived MSCs [19] that showed a native activity against MM cells in vitro in parallel with a moderate osteogenic potential in vivo in healing the MM bone lesions in SCID-rab mice. These studies, however, supported the general cytostatic property of MSCs which was apparently mild and unselective for MM cells while the bone-repairing capacity was also related to the constitutive osteoblast differentiation of cells belonging to the mesenchymal lineage as placenta-derived MSCs [19]. Further work by ourselves adopted AT-MSCs which were transduced with *TRAIL* and definitely promoted apoptosis in U-266 cells by the caspase-3 activation [30] in a similar manner as for other tumor models using these engineered MSCs [31–34]. In the present study, we preferred to transduce UC-MSCs with *TRAIL* for several reasons: large availability of MSCs from UCs; spontaneous chemotactic activity toward the tumor; and constitutive anti-MM activity by their secretome. We also used UC-MSCs since fetal MSCs are very poorly immunogenic for the minor expression of costimulatory molecules [18] while showing multipotent plasticity [35] and no tumorigenic potential [36]. In addition, UC-MSCs have been reported to show higher kinetics of proliferation and karyotypic stability in culture than AT-MSCs and BM-MSCs [37, 38].

Animal models of xenogenic tumors treated with *TRAIL*-transduced MSCs provided evidence on the effectiveness of this cell-based approach in relation to the expression of DR4/DR5 by the target tumors [16, 33, 34, 39], although several concerns regard the defective specificity since the engineered MSCs also deliver TRAIL to both tumor and normal cells, and therefore normal tissues including liver and kidney are recurrently damaged following the systemic injections of these cells [27]. On the other hand, it has been reported that, particularly in hematological malignancy including MM, based on the high expression of DR4/DR5 molecules, the soluble TRAIL isoform is also capable of inducing apoptosis in a similar manner, although less effective, than in the membrane bound form as presented by the transduced cells [40]. Thus, differentially structured viral vectors were engineered for *TRAIL* gene insertion to transduce MSCs and the full-length gene sequence codifying its soluble form has been variably inserted. However, some evidence demonstrated that the cancer cell-killing potential induced by the full-length form expressed on MSC membrane is more efficacious than that obtained by the soluble molecule [15].

Therefore, to maintain the full-length structure of the apoptotic protein and to selectively kill MM cells, we designed a vector inducing the expression of TRAIL only in the presence of cytokines secreted locally within the MM microenvironment. To this, we adopted the retroviral bicistronic vector pMIGR1 incorporating the full-length *TRAIL* gene whose expression is regulated by *pIL6*. Several cytokines such as IL-1α and IL-1β are largely secreted by MM cells within the marrow microenvironment to activate the IL-6 secretion by BM MSCs [41–43] through the stimulation of its own promoter by EGR-1 [23, 24, 44]. We reasoned that the *pIL6* insertion just before *TRAIL* within the pMIGR1 would have been able to upregulate the expression of the protein by *pIL6-TRAIL* ^*+*^ *-GFP* ^*+*^-UC-MSCs in response to IL-1α and IL-1β. Although, in the presence of a basal expression of TRAIL which was probably driven by an autocrine loop of cytokine secretion regulated by the UC-MSCs secretome [18], in our experiments we found that this structural variant of the vector significantly increased the production of TRAIL as both membrane-bound and soluble protein also in response to the conditioned medium from U-266 cells. On the other hand, the minor expansion of the osteolytic lesions in tibiae of mice treated with those cells confirmed the capacity of our vector to trigger the secretion of the apoptosis inducer in the presence of MM cells resulting in tumor cell death by activation of caspase-8 [31, 39]. This result was enough to support the efficacy of our cell-based approach against MM in mice.

The anti-MM killing of these cells was tested in our orthotopic in vivo model of MM. SCID mice were injected IT with U-266 cells to resemble the human MM model in which malignant plasma cells expand within the bone marrow and promote the bone resorption inducing osteolytic lesions [45]. Then, after developing the bone lesions, MM-bearing mice were treated with injections IC of *pIL6-TRAIL* ^*+*^ *-GFP* ^*+*^ *-*UC-MSCs and periodically investigated for the anti-MM effect as well as for their tissue distribution. By evaluating the GFP expression in several ex vivo organs, we found that the accumulation of *pIL6-TRAIL* ^*+*^ *-GFP* ^*+*^-UC-MSCs occurred in tibiae as well as in lung, heart, and renal glomeruli, thus supporting the typical tropism also to the tumor sites ascribed to MSCs [27]. However, these cells did not produce local damage in healthy tissues and they had no toxicity that could compromise the quality of life in mice. This is probably due to the low levels of both membrane and soluble TRAIL basally expressed by *pIL6-TRAIL* ^*+*^ *-GFP* ^*+*^-UC-MSCs, since the transcription of *TRAIL* is reinforced by both IL-1α and IL-1β usually abundant within the tumor sites. Such a spontaneous homing of UC-MSCs toward the myeloma tumor milieu within tibiae is apparently related to the overexpression of a number of genes such as growth factor receptor-bound 2 (*GRB2*), which are activated to promote the cell migration toward inflamed sites in response to the cell stimulation by tumor-derived chemokines [18]. Thus, in line with previous studies in different tumor models [6, 46], we interpreted the accumulation of *pIL6-TRAIL* ^*+*^ *-GFP* ^*+*^-UC-MSCs within tibiae of MM-bearing mice as an effect of their typical attraction toward the cytokine-enriched MM environment. Our result is consistent when considering that these cells migrated toward the tumor sites after the injections IC that were adopted in our animal model to avoid the potential entrapment of transduced UC-MSCs within lungs after intravenous administration.

In our model, as *pIL6-TRAIL* ^*+*^ *-GFP* ^*+*^ *-*UC-MSCs were induced in vitro to express TRAIL in response to the U-266 conditioned medium and IL-1α and IL-1β stimulation, we observed in vivo the selective pressure of the MM microenvironment to induce TRAIL at high levels. In fact, after 12 h post inoculation we observed on tibiae sections an extended apoptosis of U-266 cells by caspase-3 activation. This effect persisted up to 48 h, suggesting that the *pIL6-TRAIL* ^*+*^ *-GFP* ^*+*^-UC-MSCs exhibit a higher survival rate in bone tissue compared to other organs in which these cells are cleared faster [20], while the reduction of the bioluminescent signal of the tumor burden at different time points and even 30 days after injection supported their MM cytotoxic activity.

It has been described that placenta-derived MSCs are capable of rebuilding the MM bone lesions in mice [19]. In our model the transduced UC-MSCs also induced a partial restoration of the bone structure as shown by X-ray analysis of tibiae. Although this aspect needs to be confirmed by further work, UC-MSCs also appear capable of restoring the balance between osteoblasts and osteoclasts altered by intratibial MM expansion. The accumulation of functional MSCs within tibiae was apparently functional in interacting and stimulating the bone marrow osteoblast precursors by secreted factors that induce their differentiation into bone-building osteoblasts [20]. At the same time, UC-MSCs also restrain the osteoclast activity by secreting specific molecules [19].

## Conclusions

Our model of cytotherapy appears suitable in overcoming the drawback of the high soluble TRAIL amounts injected to induce tumor suppression, which have a short half-life when systemically infused and for which previous clinical trials failed to obtain the expected results [47]. By contrast, since *pIL6-TRAIL* ^*+*^ *-GFP* ^*+*^-UC-MSCs are committed to overexpress TRAIL only in the presence of specific cytokines secreted within the bone MM microenvironment, this cell-based therapy model would be suitable for human studies not only in controlling the marrow MM progression, but also in other osteotropic tumors since preclinical observation confirmed the biosafety of viral-transduced MSCs for TRAIL expression [48].

## Abbreviations

Ab: Antibody
AT: Adipose tissue
BM: Bone marrow
DMEM: Dulbecco’s modified Eagle’s medium
DR: Death receptor
EGR: Early growth response
FITC: Fluorescein isothiocyanate
FSC: Forward scatter
GAPDH: Glyceraldehyde-3-phosphate dehydrogenase
GFP: Green fluorescent protein
GRB: Growth factor receptor-bound
IC: Intracardially
IL: Interleukin
IT: Intratibially
MEM: Modified Eagle’s medium
MFI: Mean fluorescence intensity
MM: Multiple myeloma
Mo: Monoclonal
MSC: Mesenchymal/stromal stem cell
OD: Optical density
P: Promoter
PBS: Phosphate buffered saline
PCR: Polymerase chain reaction
PE: Phycoerythrin
photons/s: Photons per second
PI: Propidium iodide
P*IRES*: Poliovirus internal ribosome entry site
q: Quantitative
RPMI: Roswell Park Memorial Institute
SCID: Severe combined immunodeficient
SD: Standard deviation
SSC: Side scatter
TRAIL: Tumor necrosis factor related apoptosis inducing ligand
TRITC: Tetramethylrhodamine
UC: Umbilical cord
WB: Western blot
